# Epidemiology of Dermatomyositis and Other Idiopathic Inflammatory Myopathies in Northern Spain

**DOI:** 10.3390/biomedicines13102537

**Published:** 2025-10-17

**Authors:** Cristina Corrales-Selaya, Diana Prieto-Peña, David Martínez-López, Fabricio Benavides-Villanueva, Ricardo Blanco

**Affiliations:** 1 Hospital Universitario Marqués de Valdecilla, Rheumatology, 39008 Santander, Spain; c.corrales.selaya@gmail.com (C.C.-S.); diana.prieto.pena@gmail.com (D.P.-P.); david200999@hotmail.com (D.M.-L.); fabribenvillanueva@hotmail.com (F.B.-V.); 2 IDIVAL Health Research Institute of Cantabria, 39008 Santander, Spain

**Keywords:** idiopathic inflammatory myopathies, dermatomyositis, polymyositis, antisynthetase syndrome, immune-mediated necrotizing myopathy, epidemiology, prevalence, incidence rate

## Abstract

**Background/Objectives**: The epidemiology of dermatomyositis (DM) and other idiopathic inflammatory myopathies (IIMs) remains not well established, especially in the Mediterranean region. We aimed to estimate the prevalence and incidence of IIM in a well-defined population of South Europe using standardized classification criteria. **Methods**: This population-based study included all IIM patients diagnosed from January 2000 to December 2022 in Cantabria, Northern Spain. IIM diagnosis was confirmed by fulfillment of the 2017 EULAR/ACR classification criteria or, alternatively, by European Neuro Muscular Center criteria for immune-mediated necrotizing myopathy (IMNM) and Connors’ criteria for antisynthetase syndrome (ASyS). Prevalence and incidence were expressed in cases per 100,000. A literature review was also performed. **Results**: A total of 60 patients (41 women, 19 men; mean age 52.6 ± 18.8 years) were included. The prevalence of IIM was 20 cases per 100,000 population [95% CI 14.5–25.1], and the annual incidence rate was 0.9 cases per 100,000 person-years [95% CI 0.6–1.14]. A significant upward trend in IIM incidence was observed with an estimated annual percentage change of 5.74% (95% CI: 2.16%–9.44%, *p* = 0.0015). The most common subtype was DM (n = 31, 51.7%), followed by ASyS (n = 17, 24%), IMNM (n = 9, 14.6%), and polymyositis (PM) (n = 3, 4.7%). No inclusion body myositis (IBM) cases were identified. **Conclusions**: Incidence and prevalence of IIM align with prior reports. We observed an increase in IIM incidence and a shift in subtype distribution, with ASyS and IMNM becoming more frequent. These findings have clinical relevance, as each IIM subtype carries distinct prognostic and therapeutic implications.

## 1. Introduction

Idiopathic inflammatory myopathies (IIMs) are a diverse group of muscle disorders marked by inflammation, leading to progressive proximal muscle weakness and systemic manifestations [1]. The etiology of IIM is multifactorial, involving genetic predispositions, environmental triggers, and immune system dysregulation. Traditionally, IIMs, such as polymyositis (PM), dermatomyositis (DM), and inclusion body myositis (IBM), have been classified primarily based on clinical, histopathological, and electromyographic criteria [2,3]. However, the classification of IIM has evolved over the years, incorporating new myositis-specific autoantibodies (MSAs) and clinical features, leading to more refined classification schemes that integrate clinical, serological, and pathological features. The identification of antisynthetase antibodies, such as anti-Jo-1, defined the antisynthetase syndrome (ASS), associated with additional systemic manifestations like interstitial lung disease and arthritis. Similarly, the presence of autoantibodies such as anti-SRP and anti-HMGCR led to the recognition of immune-mediated necrotizing myopathy (IMNM), distinguishing it from other forms of inflammatory myopathy by its specific clinical and laboratory characteristics [4].

Although relatively rare, IIMs represent a significant cause of morbidity due to their impact on muscle function and systemic involvement. The incidence of IIMs is estimated to range from 0.2 to 2 per 100,000 person-years with prevalence from 2 to 25 per 100,000 people [5]. The variability in prevalence rates can be attributed to differences in diagnostic criteria, population demographics, and healthcare accessibility across different regions.

IIM affects all age groups, but demographic trends differ by subtype. DM, PM, and ASS are more prevalent in women [6,7,8,9], while IBM is more common in men [10,11], possibly due to hormonal influences. IMNM shows minimal gender variation [5]. DM occurs in both children and adults, whereas IBM and IMNM mainly affect individuals over 50 [12]. Some studies have shown evidence of seasonal variation [13,14] and spatial clustering [8,15] in the incidence of IIM, suggesting that environmental factors may contribute to disease development.

In recent years, improved awareness, diagnostic techniques, and development of classification criteria have contributed to a more accurate understanding of the epidemiology of IIM. The low frequency and diverse presentations of IIM, including amyopathic forms with mainly skin or lung involvement, complicate epidemiological research. Most studies are from North America and Northern Europe, with limited data from Mediterranean Europe [5].

Accurate epidemiological data on IIM are essential to understand disease burden, guide healthcare resource allocation, raise clinical awareness, and provide a foundation for future research on risk factors, prognosis, and treatment strategies.

The purpose of this study was to provide a detailed population characterization of the demographic features, clinical manifestations, laboratory findings, and therapeutic strategies in patients diagnosed with DM and other IIM in Northern Spain.

## 2. Materials and Methods

### 2.1. Design of the Study

A retrospective, population-based study was conducted, including all incident cases of IIM diagnosed between January 2000 and December 2022, in a defined healthcare area of Northern Spain. The public health system in this region is organized around a single tertiary referral center, Marqués de Valdecilla University Hospital in Cantabria.

Eligible patients were identified through a systematic review of electronic health records, hospital discharge codes, and multidisciplinary clinic lists. In order to minimize the risk of missing milder or exclusively extramuscular cases, we undertook a comprehensive case ascertainment strategy that included a thorough search of hospital-based diagnostic codes across relevant specialties (rheumatology, neurology, dermatology, pneumology, and internal medicine). In addition, we manually reviewed electronic health records for patients with suggestive codes (e.g., autoimmune skin rash and interstitial lung disease) to identify possible cases of amyopathic or hypomyopathic DM.

**Figure 1:**
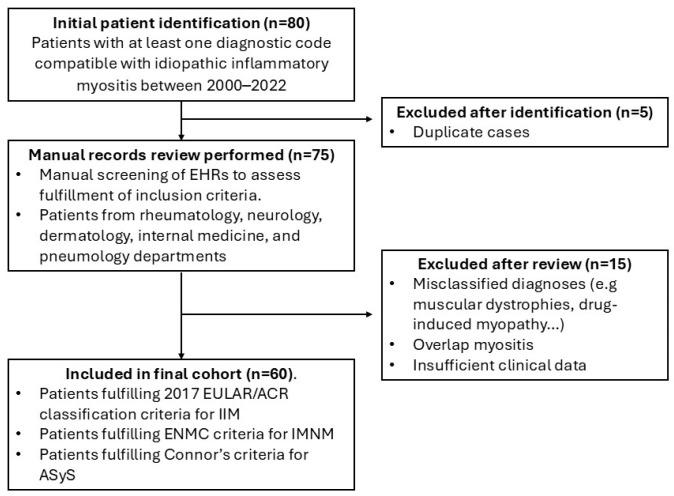
Flowchart showing the selection of patients who were included/excluded. ASyS: antisynthetase syndrome, EHR: electronic health records, ENMC: European Neuro Muscular Center, IIM: idiopathic inflammatory myositis, IMNM: immune-mediated necrotizing myopathy.

Inclusion was based primarily on fulfillment of the 2017 EULAR/ACR classification criteria for IIM. In patients with clinically compatible features but who did not meet these criteria, diagnoses were confirmed using the European Neuromuscular Center (ENMC) criteria for ENMC [16] and Connor’s criteria [17] for ASyS. Patients with overlap myositis and non-inflammatory myositis were excluded from the study to ensure a more homogeneous study population and to avoid potential confounding effects related to the coexistence of other systemic autoimmune diseases (Figure 1).

### 2.2. Outcome Variables

Clinical information was obtained from medical records. The following clinical variables were collected: age, sex, age at diagnosis, time of evolution, exposure to statins, muscle weakness, myalgia, skin involvement (Gottron’s papules, Gottron’s sign, and heliotrope rash), dysphagia, extramuscular involvement (constitutional symptoms, lung disease, arthralgias, arthritis, vasculitis, Raynaud’s phenomenon, and calcinosis), and comorbidities (Diabetes Mellitus, hypertension, dyslipidemia, chronic kidney disease, cardiovascular disease, stroke, cancer, liver disease, and osteoporosis). Laboratory parameters included creatine kinase (CK), aldolase, lactate dehydrogenase (LDH), aspartate aminotransferase (AST), alanine aminotransferase (ALT), C-reactive protein (CRP), and erythrocyte sedimentation rate (ESR). The presence of antinuclear antibodies (ANAs), myositis-specific autoantibodies (MSAs), and myositis-associated autoantibodies (MAAs), including anti-Jo-1, Mi2, TIF-1Y, NXP2, MDA5, SAE, PL-7, PL-12, EJ, OJ, HMGCoAR, SRP, KU, RO52, PM-SCL70, and PM-SCL100, was retrieved from medical records. Magnetic resonance imaging and histopathological findings of a muscle biopsy were recorded if present. Historical immunosuppressive therapies such as corticosteroids, methotrexate (MTX), azathioprine (AZA), cyclophosphamide (CFM), mycophenolate (MMF), hydroxychloroquine (HQC), infliximab (IFX), rituximab (RTX), and intravenous immunoglobulins (IVIG) were also collected.

### 2.3. Statistical Analysis

Prevalence and incidence were expressed as the number of cases per 100,000 population and 100,000 population-year, respectively. To estimate incidence rates, population data were obtained from the Cantabria Health Service’s annual reports (https://www.scsalud.es/memorias, accessed on 30 January 2023) and the National Statistics Institute (https://www.ine.es/, accessed on 30 January 2023). The annual incidence rate was calculated as the number of new IIM cases each year, relative to the population at risk for the disease in that year.

Statistical analysis was performed using R Commander for macOS, version R 4.1.2, GUI 1.77, High Sierra. All continuous variables were tested for normality, and results were expressed as mean ± SD or as median and interquartile range (IQR) as appropriate. Qualitative variables were expressed as absolute numbers and percentages (%). A Poisson regression analysis was used to evaluate the time trend in the annual number of incident IIM cases over the study period.

### 2.4. Literature Review

A literature review was conducted using PubMed and Medline covering the period from January 2000 to December 2023. The search combined terms related to idiopathic inflammatory myopathies (dermatomyositis, polymyositis, immune-mediated necrotizing myopathy, and antisynthetase syndrome) with terms related to epidemiology, incidence, and prevalence. Detailed search strings, along with inclusion and exclusion criteria, are provided in Supplementary S1.

All procedures were carried out according to the ethical standards of the approved guidelines and regulations, following the Declaration of Helsinki. This study was approved by the Institutional Review Board of Cantabria 2020.296.

## 3. Results

**Figure 2:**
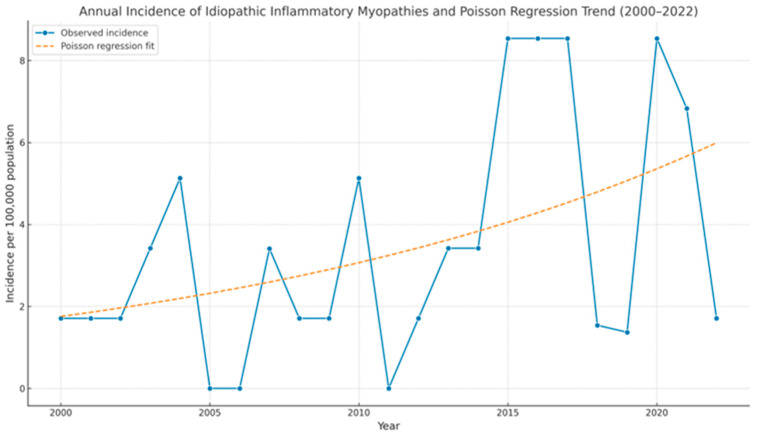
Annual incidence of idiopathic inflammatory myopathies and Poisson regression trend, 2000–2022. The blue line shows the observed annual incidence (per 100,000 population). The dashed orange line represents the predicted incidence based on Poisson regression. The model identified a statistically significant increase in incidence over time (APC = 5.74%; 95% CI: 2.16%–9.44%; *p* = 0.0015).

The main demographic, clinical characteristics, and analytical parameters of IIM patients are shown in Table 1. We identified a total of 60 IIM patients during the 2000–2022 period. The prevalence of IIM was 20 cases/100,000 [95% CI 14.5–25.1] population, and the annual incidence rate was 0.9 cases per 100,000 person-years [95% CI 0.6–1.14]. A significant upward trend in the annual number of new IIM cases was observed over the study period, with an estimated annual percentage change (APC) of 5.74% (95% CI: 2.16%–9.44%, *p* = 0.0015) according to Poisson regression analysis (Figure 2)

**Figure 3:**
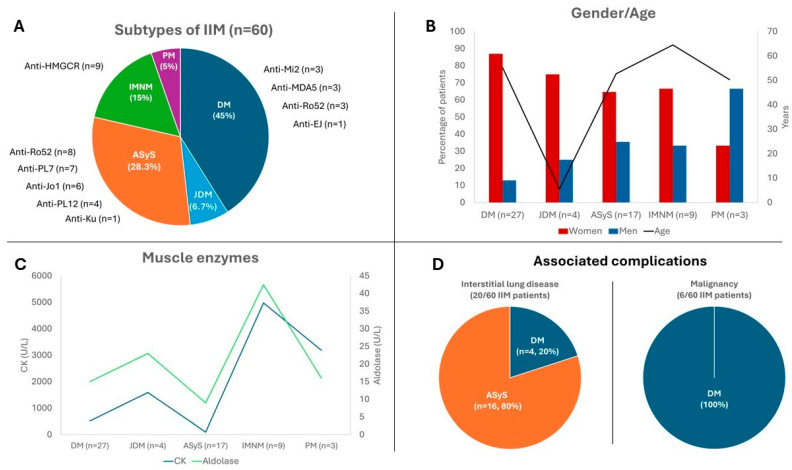
Demographic and clinical features of idiopathic inflammatory myopathies in Northern Spain. (**A**) Distribution and myositis-associated autoantibodies. (**B**) Age and sex gender distribution. (**C**) Serum creatine kinase and aldolase levels. (**D**) Associated Complications. ASyS: antisynthetase syndrome, DM: dermatomyositis, JDM: juvenile dermatomyositis, IMNM: immune-mediated necrotizing myositis, PM: polymyositis.

Considering all IIM patients, a prevalence of women (n = 41, 68.3%) was observed with a mean age at diagnosis of 52.6 ± 18.8 years and a median time from the onset of symptoms to diagnosis of 3 [0.1–10] months. The most common subtype of IIM was DM (n = 31, 51.7%), followed by ASyS (n = 17, 24%), IMNM (n = 9, 14.6%), and PM (n = 3, 4.7%). No patient with IBM was identified in our geographic region during the study period (Figure 3A).

All patients underwent antibody testing. MRI was performed in most and ASyS patients (59%), in a subset of adult DM (46%), juvenile DM (25%), and IMNM (44%) patients, and in all PM cases. Muscle biopsy was performed in the majority of patients with ASyS and necrotizing myopathy, in about half of adult DM cases, and in selected juvenile DM and PM patients (Table 1).

### 3.1. Dermatomyositis

A total of 31 DM patients were identified, being the most common subtype of IIM. Adult DM accounted for most of the patients (n = 27, 77.4%) and JDM for four (22.6%) patients.

#### 3.1.1. Adult DM

Most adult DM patients were women (n = 23, 87%), with a mean age at diagnosis of 55.2 ± 17.9 years. The incidence rate was 0.36 cases per 100,000 person-years [CI 95% 0.35–0.37], and the prevalence was 9 cases per 100,000 population [95% CI 5.6–12.3]. Among the twenty-seven adult DM patients, eight were classified as amyopathic DM (26.7%) and five as paraneoplastic DM (14.7%). The most frequent clinical manifestations in this group were skin rash (n = 26, 96.3%), Gottron’s papules (n = 22, 81.5%), muscle weakness (n = 21, 77.7%), Gottron’s sign (n = 20, 74.1%), and dysphagia (n = 11, 40.7%). Interstitial lung disease (ILD) was found in 14.8% (n = 4). The median maximum CK levels were 500 [198–1301y] IU/L. The most frequently detected myositis-specific autoantibody was anti-Mi-2 (n = 3, 11.1%) and anti-MDA5 (n = 3, 11.1%). Anti-RO52 was present in three patients (11.1%). All anti-MDA5 positive patients presented with ILD and cutaneous manifestations, but only one (33.3%) had muscle weakness. Among the patients with paraneoplastic dermatomyositis, no positive anti-TIF-1γ antibodies were identified; three patients were anti-Mi-2 positive. Nine patients had undergone MRI, all of whom presented with muscle edema, while atrophy was found only in two patients (22.2%). All patients received oral corticosteroid treatment, with a median dose of 40 ± 17.8 mg/day, and 55.6% (n = 15) required intravenous methylprednisolone boluses. The most frequent glucocorticoid-sparing agents in decreasing order of frequency were AZA (n = 14, 51.9%), HQC (n = 13, 48.1%), RTX (n = 11, 40.7%), and MTX (n = 10, 37.0%). Seventeen (72.7%) patients received at least one cycle of IVIG (Table 2)

#### 3.1.2. Juvenile DM

Juvenile DM accounted for 22.6% (n = 4) of the cases; most of them were female (n = 3, 75%), with a mean age at diagnosis of 5.5 ± 1.5 years. The incidence rate was 0.6 cases per million person-years [CI 95% 0.55–0.65], and the prevalence was 1 case per 100,000 population [95% CI 0.1–2.6]. All patients had cutaneous involvement, most frequently presenting with skin rash (n = 4, 100%) and proximal muscle weakness. Additionally, three patients (75%) had dysphagia, and two patients (50%) had calcinosis. No myositis-specific autoantibodies were identified in this group of patients. All patients required treatment with oral corticosteroids, with a mean dose of 30 ± 10 mg/day of prednisone; however, only one patient required intravenous corticosteroids. As steroid-sparing agents, all patients received MTX, and two of them (50%) received at least one course of IVIG (Table 2).

### 3.2. Antisynthetase Syndrome

This subgroup represented the second most common type of IIM in this series, comprising 17 patients (24%). Of these, 11 were female (64.7%), with a mean age at diagnosis of 52.7 ± 11.7 years. The incidence rate was 0.26 cases per 100,000 person-years [95% CI: 0.24–0.27], and the prevalence was 5 cases per 100,000 population [95% CI: 4.7–6.6]. ILD and Raynaud’s phenomenon were present in 94.1% and 52.9% of patients, respectively. Myalgia occurred in 26.7% (n = 4) and proximal weakness in 26.7% (n = 3) of cases. The median CK levels were 100 [40–885y] IU/L. All patients exhibited antisynthetase autoantibodies, with the most frequent being anti-PL7 (n = 7, 41.2%), followed by anti-Jo-1 (n = 6, 35.3%) and anti-PL12 (n = 4, 23.5%). Concerning myositis-associated autoantibodies, the most frequent was anti-Ro52 (n = 8, 47%), followed by anti-Ku (n = 1, 5.9%). All patients received oral corticosteroids, with a median dose of 30 [25.6–40.0] mg/day. A third of the patients received methylprednisolone bolus therapy. Notably, 70.6% of patients were treated with RTX. The conventional immunosuppressive agents used, in decreasing order of frequency, were MMF (n = 9, 52.9%), AZA (n = 8, 58.8%), and CFM (n = 4, 23.5%). Only one patient (5.9%) received IVIG (Table 2).

### 3.3. Immune-Mediated Necrotizing Myopathy

The third most frequent subgroup of IIM was IMNM of the patients (66.6%) who received IVIG (Table 2), accounting for 14.6% (n = 9) of cases. Similar to the previous groups, most patients were women (n = 6, 66.6%), with a mean age at diagnosis of 64.5 ± 7 years. The incidence rate was 0.14 cases per 100,000 person-years [CI 95% 0.13–0.15], and the prevalence was 3 cases per 100,000 population [CI 95% 2.1–3.8]. All patients had dyslipidemia and were receiving statin treatment at the time of diagnosis. Muscle weakness was present in all patients, and dysphagia was observed in three patients (33.3%). The median maximum CK levels were 4977 [3273–9271] IU/L. Anti-HMGCoR autoantibodies were present in all cases, while no anti-SRP autoantibodies were detected. Seven patients (77.8%) received oral corticosteroids, with a median dose of 5 [2.5–6.25] mg/day. The most frequently associated immunosuppressive agents were RTX (n = 4, 44.4%), AZA (n = 3, 33.3%), and MTX (n = 2, 22.2%).

### 3.4. Polymyositis

The least frequent subgroup in our cohort was PM, with only three (4.7%) patients (one woman/two men), with a mean age of 50.3 ± 11.0 years. The incidence rate was 0.45 cases per million person-years [CI 95% 0.43–0.47], and the prevalence was 1 case per 100,000 population [CI 95% 0.5–1.5]. The most frequent manifestations were muscle weakness (n = 2, 66.6%) and myalgias (n = 2, 66.6%), followed by non-specific systemic manifestations (n = 1, 33.3%) and Raynaud’s phenomenon (n = 1, 33.3%). None of the patients had skin lesions or cancer. No antibodies associated with inflammatory myopathies were detected in any patient. All patients received oral corticosteroids, with a median dose of 40 [40–45] mg/day. The most common glucocorticoid-sparing agents used were MTX (n = 2, 66.6%) and AZA (n = 2, 66.6%). One patient (33.3%) received IVIG (Table 2).

## 4. Discussion

The present study reports the first epidemiological data on DM and other IIMs in Northern Spain, identifying 60 cases diagnosed from 2000–2022, with a prevalence of 20 per 100,000 and an incidence of 0.9 per 100,000 person-years. Additionally, we found a significant progressive increase in the annual incidence of IIMs with an estimated APC of 5.74% (95% CI: 2.16%–9.44%, *p* = 0.0015). This trend may reflect multiple factors, including a true increase in disease occurrence due to environmental or genetic influences, as well as improved disease recognition, broader antibody testing, and more frequent use of advanced diagnostic techniques such as MRI. Greater clinical awareness and increased availability of specialized care may also contribute.

The development of new myopathy classification criteria has led to a change in the distribution of the different subgroups of myopathies. The Bohan and Peter classification, which has been in use for many years, includes patients with IMNM and ASyS within the PM subgroup. This renders direct comparisons with previous studies more complex and is likely to result in an underestimation of the incidence and prevalence reported for these specific myopathy subtypes. In our cohort, the largest subgroup was DM (51.7%), followed by ASyS (24%), IMNM (14.6%), and PM (4.7%). Notably, we have no patients with IBM. Our study enhances understanding of IIM epidemiology by subgroup, particularly IMNM and ASyS, for which data are limited (Table 3a,b).

Our study found a higher proportion of women with IIMs, except in the PM subgroup, though a small cohort size limits strong conclusions (Figure 3B). Excluding JDM, the mean age at diagnosis ranged from 50 to 60 years, aligning with prior studies [37]. The IMNM group represented the oldest age cohort. This subgroup also demonstrated a higher prevalence of cardiovascular risk factors (Table 1), which may be relevant to its pathophysiology.

Regarding the clinical features of each myopathy subgroup (Table 1), our findings are consistent with those previously reported [4,37]. Our data also demonstrate distinct patterns of muscle enzyme changes across different myopathy subgroups. CK and aldolase levels were elevated to a greater extent in IMNM, whereas ASyS showed a less pronounced increase (Figure 3C).

We also analyzed the prevalence of ILD and malignancy (Figure 3D). The prevalence of ILD in myositis has been described to range from 19.9% to 42.6%, particularly in association with specific antibodies such as anti-Jo1, PL-7, and PL-12 [38]. In our cohort, one-third of the patients (20/60) developed ILD, with 80% belonging to the ASS subgroup and 20% to DM. None of the patients with JDM, IMNM, and PM developed ILD during the study period. In the ASyS group, the antibodies most frequently associated with ILD were anti-Ro52 (n = 8, 47.1%), anti-Jo1 (n = 6, 35.3%), and anti-PL-7 (n = 6, 35.3%), followed by anti-PL-12 (n = 4, 23.5%) and anti-Ku (n = 1, 5.9%). In the adult DM group, anti-Mi2 (n = 3, 11.1%), anti-MDA5 (n = 3, 11.1%), and anti-Ro52 (n = 3, 11.1%) were the most frequently associated autoantibodies. The association of malignancies with myositis has also been widely described in the literature [37], with great variability between cohorts. In our study, 10% of patients developed malignancies (Figure 3D), all belonging to the DM group. Colorectal cancer was the most frequent (n = 2, 33.2%), followed by ovarian (n = 1, 16.6%), breast (n = 1, 16.6%), pharynx (n = 1, 16.6%), and cholangiocarcinoma (n = 1, 16.6%).

In terms of therapeutic strategies, a large proportion of patients in all myopathy subgroups received IVIG, except for those in the ASS subgroup, where RTX was the most frequently used immunosuppressive agent (Table 2).

The findings of this study are of considerable value despite inherent limitations. Although the design was population-based and encompassed a 23-year period, the sample size remains relatively small, reflecting both the rarity of IIM and the limited population of the healthcare region. Data from other regions of Spain are currently limited, and therefore, it is not possible to determine whether the observed increase in IIM reflects a local phenomenon or a nationwide trend. As a result, the generalizability of our findings to other populations or healthcare settings may be limited, and interpretations should be made with caution. We acknowledge that some very mild or atypical cases seen outside hospital settings or without coding might have escaped detection. However, it is important to note that this center provides specialized rheumatology, neurology, dermatology, and internal medicine services for the entire region. This structure minimizes the likelihood of missing cases within the covered population. This study benefits from a geographically homogeneous cohort, which ensures consistency in environmental factors and healthcare practices. Furthermore, uniform application of classification criteria enhances the reliability of the study’s findings. However, we are aware that the retrospective nature of this study and the evolution of diagnostic standards over the study period may have led to misclassification or under-recognition of certain subtypes, particularly in earlier years.

The absence of IBM cases over the 23-year study period was unexpected, given the demographic structure of the population and the known epidemiology of IBM. To address this, we conducted targeted searches within neurology and rheumatology records, neuromuscular clinic logs, and the institutional muscle biopsy registry, focusing on patients aged ≥ 50 years with suspected inflammatory or degenerative myopathies. No confirmed cases of IBM were identified. Nonetheless, we acknowledge the possibility of under-recognition or misclassification, particularly during the earlier years of the study when awareness and diagnostic criteria were less established. This represents a limitation of our study.

Therapeutic data were extracted from clinical records and may be affected by incomplete documentation or lack of standardization in treatment reporting, particularly regarding timing, duration, and rationale for therapeutic choices. Although we attempted to provide a descriptive overview of treatment patterns, these findings should be interpreted as exploratory.

Despite these limitations, this study offers valuable insights into the long-term epidemiology, clinical spectrum, and management of IIM in a real-world, population-based setting.

## 5. Conclusions

In conclusion, this study provides novel information regarding the epidemiology of IIM in the Mediterranean or Southern European region, where existing data are limited. The incidence, prevalence, and age/sex distribution are similar to those described in previous studies. It is noteworthy that an increasing incidence of IIM, along with a shift in the distribution of subgroups, was found. Specifically, the frequencies of ASyS and IMNM have increased, while PM diagnoses have declined. ILD and malignancies were identified as relevant comorbidities, observed in 33.3% and 10% of patients, respectively. To improve the current understanding of IIM, further epidemiological studies applying recently updated classification criteria across diverse geographical settings are warranted.

## Figures and Tables

**Table 1:** Demographic, clinical, and laboratory data of juvenile and adult dermatomyositis, antisynthetase syndrome, immune-mediated necrotizing myopathy, and polymyositis.

	Adult DM (n = 27)	Juvenile DM (n = 4)	ASyS (n = 17)	IMNM (n = 9)	PM (n = 3)
Demographic features					
Sex (female/male), n (%)	23 (87%)/4 (13%)	3 (75)/1 (25)	11 (64.7)/6 (35.3)	6 (66.6)/3 (33.3)	1 (33.3)/2 (66.6)
Age at diagnosis, mean ± SD	55.2 ± 17.9	5.5 ± 1.5	52.7 ± 11.7	64.5 ± 7	50.3 ± 11.0
Duration of symptoms after diagnosis (months), mean ±SD	0 [0–4.8]	0 [0–2]	6 [6–12]	4 [3–5]	18.0 [15–21]
Dyslipidemia, n (%)	9 (33.3)	0	4 (23.5)	9	1 (33.3)
Statin exposure, n (%)	4 (14.8)	0	4 (23.5)	9	1 (33.3)
Arterial Hypertension, n (%)	7 (25.9)	0	1 (5.9)	7	1 (33.3)
Type 2 DM, n (%)	4 (14.8)	0	0	7	0
Clinical manifestations					
Muscle weakness, n (%)	21 (77.8)	4 (100)	4 (28.6)	9 (100)	2 (66.6)
Myalgia, n (%)	9 (33.3)	2 (50)	4 (28.6)	4 (44.4)	2 (66.6)
Dysphagia, n (%)	11 (40.7)	3 (75)	2 (11.8)	3 (33.3)	0
Skin rash, n (%)	26 (96.3)	4 (100)	3 (17.6)	0	0
Gottron’s papules, n (%)	22 (81.5)	2 (50)	1 (5.6)	0	0
Gottron’s sign, n (%)	22 (81.5)	2 (50)	0	0	0
Heliothrope rash, n (%)	8 (29.6)	2 (50)	0	0	0
Raynaud phenomenon, n (%)	3 (11.1)	0	9 (53)	0	1 (66.6)
Malignancy, n (%)	6 (22.2)	0	0	0	0
Interstitial Lung Disease, n (%)	4 (14.8)	0	16 (94)	0	0
Arthritis, n (%)	7 (25.9)	0	5 (29.4)	0	0
Calcinosis, n (%)	4 (14.8)	2 (50)	0	0	0
Laboratory tests					
CK, median [IQR]	516 [186–1217]	1591 [946–2027]	100 [40–885]	4977 [3273–9271]	3158 [1807–3629]
Aldolase, median [IQR]	15 [10–23]	23 [21.5–24]	9 [9–16]	42.5 [26.5–63]	16 [11–53]
Anti-MDA5, n (%)	3 (10)	0	0	0	0
Anti-Mi2, n (%)	3 (10)	0	0	0	0
Anti-TIF-Y, n (%)	0	0	0	0	0
Anti-JO1, n (%)	0	0	6 (35.3)	0	0
Anti-PL7, n (%)	0	0	7 (41.2)	0	0
Anti-PL12, n (%)	0	0	4 (23.5)	0	0
Anti-EJ, n (%)	1 (3.3)	0	0	0	0
Anti-HMGCR/SRP, n (%)	0	0	0	9 (100)/0	0
Anti-RO52, n (%)	3 (10)	0	8 (47.0)	0	0
Anti-Ku, n (%)	0	0	1 (5.9)	0	0
Anti-PM-SCL100, n (%)	0	0	0	0	1 (33.3)
Imaging procedures					
Inflammatory findings on MRI, n/N (%)	9/11 (82)	1/1 (100)	10/10 (100)	4/4 (100)	2/3 (66.6)
Biopsy					
Muscle biopsy, n (%)	9 (33.3)	2 (50)	10 (58.8)	9 (100)	1 (33.3)

**Table 2:** Treatment strategies in juvenile and adult dermatomyositis, antisynthetase syndrome, immune-mediated necrotizing myopathy, and polymyositis.

	Adult DM (n = 27)	Juvenile DM (n = 4)	ASyS (n = 17)	IMNM (n = 9)	PM (n = 3)
Intravenous corticosteroids, n (%)	15 (55.6)	1 (25)	5 (29.4)	0 (0)	0 (0)
Oral corticosteroids, n (%)	27 (100)	4 (100)	17 (100)	7 (77.8)	3 (100)
Hydroxychloroquine, n (%)	13 (48.1)	0 (0)	0 (0)	0(0)	0 (0)
Azathioprine, n (%)	14 (51.9)	0 (0)	8 (58.8)	3 (33.3)	2 (66.6)
Methotrexate, n (%)	10 (37)	4 (100)	4 (23.5)	2 (22.2)	2 (66.6)
Mycophenolate, n (%)	0 (0)	0 (0)	9 (52.9)	0 (0)	0 (0)
Cyclophosphamide, n (%)	0 (0)	0 (0)	4 (23.5)	0 (0)	0 (0)
Rituximab, n (%)	11 (40.7)	0 (0)	13 (76.5)	4 (44.4)	0 (0)
Intravenous immune-globulins, n (%)	17 (72.7)	2 (50)	1 (5.9)	6 (66.6)	1 (33.3)

**Table 3:** (**a**) Literature review of published articles on the epidemiology of dermatomyositis. (**b**) Literature review of published articles on epidemiology of antisynthetase syndrome, immune-mediated necrotizing myopathy, inclusion body myositis, and polymyositis.

(a)						
Author, Year (Ref)	Country, Region	Study Period	Diagnosis Criteria	Number of Cases	Prevalence	Incidence (Per 100,000 People Except Where Noted)
Adult DM						
Bendewald M et al., 2010 [18]	USA	1976–2007	Adapted from Gerami et al.	20 DM	21.4	0.96
Bolender C et al. 2022 [19]	USA	2005–2019	Coded registration	679 DM		0.8
Kronzer et al. 2023 [20]	USA	1995–2019	EULAR/ACR 2017	29 DM	13.0	1.1
Balci M et al. 2017 [21]	Turkey	2004–2014	Bohan and Peter	23 DM	3.2	0.37
Present study, 2025	Spain	2000–2022	EULAR/ACR 2017	27 DM	9.0	0.36
Juvenile DM						
Mendez et al. 2003. [22]	USA	1994–1999	Bohan and Peter	395 JDM		0.32
Concannon C et al. 2021. [23]	New Zealand	2000–2020	Bohan and Peter	31 JDM		0.24
Moegle C et al. 2020. [24]	France	2000–2015	Bohan and Peter or EULAR/ACR 2017	16 JDM	3.78	0.27
Enders et al. 2011 [25]	Switzerland	1997–2010	Expertise Criteria	13 JDM	0.35	
Symmons et al. 1995 [26]	UK	1992–1993	Bohan and Peter	48 JDM		0.19
Present study, 2025	Spain	2000–2022	EULAR/ACR 2017 criteria	4 JDM	1.0	0.006
(**b**)						
**Author, Year (Ref)**	**Country, Region**	**Study Period**	**Diagnosis Criteria**	**Number of Cases**	**Prevalence**	**Incidence**
ASyS						
Coffey et al. 2021 [27]	USA	1998–2019	Salmon’s Criteria	13 ASyS	9.0	0.56
Present Study, 2025	Spain	2000–2022	Connor’s Criteria	17 ASyS	5.0	0.26
IBM						
Felice et al. 2001 [28]	USA	1992–2000	Griggs Criteria	35 IBM	2.9	
Shelly et al. 2021 [29]	USA	2010–2019	ENMC 2011	21 IBM	19.2	0.47
Phillips et al. 2000 [30]	Australia	1988–1998	Griggs Criteria	17 IBM	3.53	
Lefter et al. 2017 [31]	Ireland	1990–2013	MRC	149 IBM	11.7	
Badrising et al. 2000 [10]	Netherlands	1982–1999	ENCM	128 IBM	3.2	0.25
Dobloug et al. 2015 [11]	Norway	2003–2012	ENMC 2011	95 IBM	3.3	0.2–0–6
Lindgren et al. 2022 [32]	Sweden	1985–2017	ENMC 2011	128 IBM	3.2	0.25
Present Study, 2025	Spain	2000–2022	ENMC	0 IBM		
IMNM						
Shelly S et al. 2022 [33]	USA	1999–2019	ENMC Criteria	7 IMNM	1.9	0.59
Prieto-Peña et al. 2022 [34]	Spain	2016–2021	ENMC Criteria	8 IMNM	3.0	0.6
Present Study 2025	Spain	2000–2022	ENMC Criteria	9 IMNM	3.0	0.14
PM						
Araki et al. 1987 [35]	Japan	1977–1982	Walton-Adams Criteria	27 PM	5.0	
Radhakrishnan et al. 1987 [36]	Libya	1983–1985	DeVere and Bradley Criteria	13 PM		0.84
Present Study 2025	Spain	2000–2022	EULAR/ACR 2017 Criteria	3 PM	1.0	0.45

## Data Availability

The data underlying this article will be shared on reasonable request to the corresponding author.

## Supplementary Materials

The following supporting information can be downloaded at: https://www.mdpi.com/article/10.3390/biomedicines13102537/s1.
