# Supplementary material for: Epidemiology of Dermatomyositis and Other Idiopathic Inflammatory Myopathies in Northern Spain
Source: Biomedicines. 2025 Oct 17;13(10):2537. doi: 10.3390/biomedicines13102537 (PMC12562039; doi:10.3390/biomedicines13102537)
Supplement: Supplementary file 1 [file biomedicines-13-02537-s001.zip › biomedicines-3884842-supplementary.pdf]

Supplementary 1. Literature search strategy and selection criteria

Search string: ("idiopathic inflammatory myopathies" OR "dermatomyositis" OR "polymyositis" OR "immune-mediated necrotizing myopathy" OR "antisynthetase syndrome") AND ("epidemiology" OR "incidence" OR "epidemiology")

Time frame: January 2000 – December 2023

Inclusion criteria:

- Original articles, systematic reviews, or meta-analyses.
- Studies including patients with idiopathic inflammatory myopathies (dermatomyositis, polymyositis, immune-mediated necrotizing myopathy, antisynthetase syndrome).
- Studies reporting epidemiology features.

Exclusion criteria:

- Case reports or small case series (<30 patients).
- Studies exclusively on overlap myositis or myopathies secondary to other systemic diseases.
- Articles not in English.

The selection process was conducted in two stages: initial screening by title and abstract, followed by full-text evaluation. Included studies were analyzed and synthesized according to myositis subtype.
